# Studies on the Regulatory Roles and Related Mechanisms of lncRNAs in the Nervous System

**DOI:** 10.1155/2021/6657944

**Published:** 2021-03-13

**Authors:** Zijian Zhou, Dake Qi, Quan Gan, Fang Wang, Bengang Qin, Jiachun Li, Honggang Wang, Dong Wang

**Affiliations:** ^1^The Seventh Affiliated Hospital of Sun Yat-sen University, Shenzhen, Guangdong, China; ^2^Memorial University of Newfoundland School of Medicine, Newfoundland and Labrador, Canada; ^3^Union Hospital of Huazhong University of Science and Technology Shenzhen Hospital, Shenzhen, Guangdong, China; ^4^The First Affiliated Hospital of Sun Yat-sen University, Guangzhou, Guangdong, China

## Abstract

Long noncoding RNAs (lncRNAs) have attracted extensive attention due to their regulatory role in various cellular processes. Emerging studies have indicated that lncRNAs are expressed to varying degrees after the growth and development of the nervous system as well as injury and degeneration, thus affecting various physiological processes of the nervous system. In this review, we have compiled various reported lncRNAs related to the growth and development of central and peripheral nerves and pathophysiology (including advanced nerve centers, spinal cord, and peripheral nervous system) and explained how these lncRNAs play regulatory roles through their interactions with target-coding genes. We believe that a full understanding of the regulatory function of lncRNAs in the nervous system will contribute to understand the molecular mechanism of changes after nerve injury and will contribute to discover new diagnostic markers and therapeutic targets for nerve injury diseases.

## 1. Main Text

Long noncoding RNAs (lncRNAs) are noncoding RNAs (ncRNAs) with a lack of significant open reading frames (ORFs) and length more than 200 nt. lncRNAs not only play a role in the growth and development of nervous system but also play a significant role in the process of nervous system injury and subsequent repair. lncRNAs affect the growth and development of the nervous system in time order and space by regulating the expression of some significant coding genes and also participate in the functional execution of the nervous system. At the same time, the expression of lncRNAs is closely related to the process of nervous system regeneration. Studies have indicated that lncRNAs are involved in the growth, development, and repair of the central and peripheral nervous systems. As a result, in-depth study of lncRNA function and mechanism of action in the nervous system will greatly enhance our understanding of nervous system development, function, and the mechanism of disease occurrence and also provide new ideas for the treatment of some diseases. This review will describe in detail the relevant research progress on the role of lncRNAs in the nervous system.

## 2. Types and Basic Functions of lncRNAs

As human genome sequence has been leaked in 2001, although there are about 90% in eukaryotic genomes transcribed, only about 1% to 2% of the genes are encoding proteins. This indicates that a large number of RNA molecules are ncRNAs [1]. The rest of the noncoding part has been considered useless transcriptional noise in the past. However, in recent decades with the development of high-throughput technology as well as the human genome sequencing, various non-protein-coding DNAs and noncoding RNAs were rediscovered. Okazaki et al. first described the existence of lncRNAs on the length of the mouse cDNA library in the process of the large-scale sequencing [2]. Subsequent researches have shown lncRNA morphological diversity, including the length of the long chain which is more than 10 KB lncRNAs and shows cricoid circRNAs. lncRNAs exist in a variety of species, including animal plant yeast prokaryotes, even virus. However, lncRNAs between species are less conservative and usually have low expression [3]. Lots of evidence show that lncRNAs play a vital role in various significant biological processes [4]. Therefore, the functional and biological characteristics of lncRNAs make it an interesting and significant research subject.

### 2.1. Types of lncRNAs

With the progress of transcriptome gene sequencing, the gene sequences and mechanism of action of various lncRNAs are constantly revealed, and there are also many classification methods for lncRNAs from different perspectives. For example, lncRNAs are classified according to their positions relative to protein-coding genes, according to their positions in specific DNA regulatory elements and loci, and according to their subcellular location or origin [5]. In this review, we mainly introduce in detail the classification based on the association of lncRNAs with specific biological processes.

#### 2.1.1. Hypoxic Induction-Related lncRNAs

Studies have indicated that a large number of lncRNAs are involved in cell damage resistance and ischemia/hypoxia/reperfusion injury [6]. For example, in the hypoxia environment, the expression of lncRNA-H19, the gene product of H19, is upregulated in response to the increased activity of hypoxia induction factor (HIF), which promotes the production of oxygen free radicals and further damages cells [4, 7], while lncRNA-ROR alleviates hypoxia-triggered damage by downregulating miRNA-145 in H9c2 cells [8].

#### 2.1.2. Senescence-Related lncRNAs

In the process of cellular senescence regulation, a large number of lncRNAs are expressed, which play a role in both promoting senescence and delaying senescence [9]. lncRNA HOTAIR upregulates autophagy to promote apoptosis and senescence of nucleus pulposus cells [10]. lncRNAs RP11-670E13.6 interact with hnRNPH and delay cell senescence by sponging microRNA-663a [11].

#### 2.1.3. Stress-Induced Related lncRNAs

Cells in the oxidative stress state will reduce the number of normal mRNA transcription, but out of a large number of transcription lncRNAs and si-lncRNAs and abundant accumulation in the cells to adapt to the stress state of cellular [12]. FILNC1 (FoxO-induced long noncoding RNA1) in renal cancer cells is an lncRNA induced by energy stress of FoxO transcription factor, and its deletion reduces the apoptosis induced by energy stress, significantly promoting the development of renal tumor [13].

#### 2.1.4. Nonannotated Stem Cells Transcribe lncRNAs

lncRNAs play a significant role in pluripotent state maintenance, especially in transcripts derived from nuclear transcription and reverse transcription transposons. L1s and solitary long terminal repeat- (LTR-) derived transcription constitutes the complexity of the stem cell nuclear transcriptome [14]. Some LTR-derived transcripts are associated with enhancers and may be involved in pluripotent maintenance [15]. Studies indicated that deep sequencing transcriptome analysis of mammalian stem cells has determined that unannotated stem cell transcripts (NAST) are significant for maintaining the pluripotency of stem cells [15].

#### 2.1.5. Disease-Specific Transcripts

With the development of clinical and diagnostic studies, more and more disease-specific lncRNAs have been detected, and the disease-related specificity of these lncRNAs is used to observe the development status and prognosis of diseases. For example, prostate cancer-related transcription (PCAT) PCAT1 has been proved to play a significant role in the biological function of prostate cancer [16].

### 2.2. The Basic Functions of lncRNAs

lncRNAs influence the transcriptional splicing, translation output, import, and stability of mRNA. Previous studies have indicated that lncRNAs play a recruitment role of transcription factors, act as transcriptional coactivators, or serve as protein scaffoldings through their interactions with transcription initiation sites [17].

lncRNAs may also act as bait to trap transcription factors, thus limiting their binding ability to DNA binding sites [18].

lncRNAs have been indicated to participate in the formation and function of chromatin-circulating nucleosomes [19].

lncRNAs also regulate mRNA processing maturity and stability by regulating mRNA splicing, inhibiting translation, or inhibiting miRNA binding sites on mRNAs by sponge action [18]. According to the definition of lncRNA, we consider circRNA as a kind of lncRNA with special structure. circRNAs are formed by reverse splicing of upstream 3′ end and downstream 5′ end, which is highly stable in cells and resistant to exogenous degradation [20]. They bind to a large number of miRNA binding sites through endogenous competition, inhibit the activity of corresponding miRNA, and elevate the expression of its downstream molecules [21]. It is highly consistent with the mechanism of lncRNA acting on miRNA.

Some lncRNAs also encode small peptides. These lncRNAs are used as bifunctional transcripts, that is, as lncRNAs or as protein translation templates [22, 23].

lncRNAs regulate protein and transcript transport and shuttling. The result of this process is that lncRNAs are associated with many biological processes, including cell cycle regulation of pluripotent retrotransposon silencing and telomere elongation in stem cells [24].

## 3. The Regulatory Role of lncRNAs in the Growth, Development, and Various Diseases of the Advanced Central Nervous System

lncRNAs' role in central nervous cell life has gained wide attention and research; many results showed that lncRNAs could regulate various cell pathways involved in the differentiation, development, and pathology of central nerve cells (including hypoxia ischemia change tumor regression). Different from the peripheral nerve injury, the central nerve is difficult to regenerate and be repaired after injury; thus, related lncRNAs also received special attention in the process of repairment and regeneration of the central nerve after injury.

### 3.1. The Regulatory Role of lncRNAs in the Growth and Development of the Advanced Central Nervous System

In recent years, the mechanisms of lncRNAs in neural development have been gradually revealed; many studies have confirmed the involvement of lncRNAs in the differentiation of neural stem cells or the development of central nerve cells. For example, Pnky is an 825 nt lncRNA. It is expressed in vitro and in vivo in neural stem cells (NSCs) and transregulate the development of the neocortex [25]. PTPB is a splicing factor that plays a key role in normal development and brain tumor. During the differentiation of neurons, the expression of PTBP1 reduced and the expression of PTBP2 increased [26]. Pnky interacts with PTBP1 and regulates the neurogenesis of embryonic and postnatal brain neural stem cells, and there is no evidence for specific interactions between Pnky and PTPB2 or other nuclear RNA binding proteins such as HNRNPK and ELAVL1 [27].

Researches have indicated that lncRNA-Evf2 is the key to promote the differentiation of the GABAergic cells. Evf2 recruits transcription factors DLX and MECP2 into key DNA regulatory elements in the intergenomic region of DLX 5/6, regulating the expression of Dlx5, Dlx6, and GAD67 [28]. Studies have indicated that the expression level of GAD67 protein in mice with Evf2 dysfunction is reduced and the number of GABA-mediated neurons in the early hippocampus and dentate gyrus decreases after birth [29]. Levels of GABA intermediate neurons and GAD67 RNA in the hippocampus of Evf2 returned to normal in adults, but failed to improve symptoms of reduced synaptic inhibition [30].

In addition to participating in the growth and differentiation of various neurons in the brain, lncRNAs are also significant for the formation and function of nerve cells in various receptors, including various nerve cells such as optic nerve and olfaction nerve. lncRNA Tug1 regulates the growth and development of retinal photoreceptor cells and regulates retinal pigment cells [31]. Tug1 deletion may result in loss or malformation of the extracellular segment of the photoreceptor [32]. lncRNA Six3OS is expressed together with the homologous domain factor Six3, which plays a key role in the development of mammalian eyes and regulates the cells of the retina at the early stage of eye formation [33]. However, Six3OS does not regulate the expression level of Six3; instead, Six3OS directly combines known transcription-assisted regulators of Six3 and histone-modifying enzymes, thus playing a role as an RNA-based transcription scaffold [34]. KAP1 is a significant epigenetic regulatory protein that interacts with chromatin-binding proteins to control the formation of heterochromatin and inhibits gene expression at autosomal sites. Pavlaki et al.'s study has indicated that lncRNAs Paupar and KAP1 are both regulators of olfactory bulb neurogenesis in vivo [35, 36]. lncRNA Paupar forms ribonucleoprotein complexes containing KAP1 and PAX6 transcription factors, which, if dysfunctional, will destroy the olfactory bulb neurogenesis [35, 37]. Pax6 plays a key role in the generation of retinal neonate neurons and in the control of the differentiation of various late neuron cell types [38].

### 3.2. The Regulatory Role of lncRNAs in Advanced Central Nervous System Diseases

The role of lncRNAs is not only reflected in the growth and development of nerve cells, but more and more studies have revealed the importance of lncRNAs in various central nervous system diseases. NRON is an lncRNA that mediates the cytoplasmic to nuclear transport of NFAT transcription factors [39]. In animal models, when NRON was removed from the regulation of DSCR1 and DYRK1A genes on chromosome 21, the expressions of DSCR1 and DYRK1A were upregulated, leading to the decrease of NFATc activity, which eventually resulted in characteristics of Down syndrome (DS) [40]. This process indicated that there was a potential link between NRON activity and the pathophysiology of DS.

lncRNA BACE1 is used as a potential biomarker for the diagnosis of Alzheimer's disease (AD). BACE1 contains 9 exons and is a candidate gene for sporadic AD [41]. Studies have indicated that the single nucleotide polymorphism of exon 5 of BACE1 gene is related to the occurrence of AD, and the improvement of BACE1 activity leads to the occurrence of AD. However, its specific potential mechanism is not yet clear [42].

In addition, the coexpression module of lncRNA DGCR5 has been confirmed to be associated with schizophrenia (SCZ). Studies have indicated that DGCR5 expression in the brain tissue of SCZ patients is significantly reduced compared with that of normal individuals [43]. DGCR5 was found to be the only CNV lncRNAs in the coexpression module of neurons downregulated in the postmortem brain tissue of SCZ autism and bipolar disorder patients [44]. These findings suggest that DGCR5 may increase the risk of SCZ through its regulatory effect on coexpressed SCZ-related genes.

Parkinson's disease is one of the most common diseases of the nervous system. A study demonstrated the functional relevance of the HOX transcript antisense intergenic RNA (HOTAIR)/microRNA-221-3 (miR-221-3p)/neuronal pentraxin II (NPTX2) axis in the process of dopaminergic neuron autophagy [45]. HOTAIR binds to miR-221-3P and improves the expression of NPTX2, thus reducing cell activity and enhancing the autophagy ability of dopaminergic neurons, while silenced HOTAIR may save the death of dopaminergic neurons by downregulating the NPTX2 gene mediated by miR-221-3p by inhibiting the autophagy of dopaminergic neurons in the substantia nigra dense region of mice [45, 46].

lncRNA is also confirmed to be widely involved in the process of traumatic brain injury (TBI). The five lncRNAs that were most significantly upregulated in TBI were N333955, n332943, N335470, ENST00000384390, and N341115. Tcons_00018733-xloc_008489, OTTHUMT00000076953, NR029967, ENST00000433249, and N381234 are the five most significantly downregulated lncRNAs [47, 48].

Brain tumors have received more attention in recent years. As a common malignant tumor of the nervous system, glioma is gradually attached importance to the association with lncRNAs. Studies have demonstrated that during the occurrence of gliomas, significant changes in the expression levels of many lncRNAs are observed, such as HOTAIR1, BDNF-ASBDNF-AS, CCAT2, CRNDE, MALAT1, TUG1, and PART1 [49–52]. Therefore, it demonstrates that the mechanism research of lncRNAs will be critical in the occurrence of gliomas and new targets for subsequent treatment. Among them, BDNF-AS is a naturally conserved lncRNA that inhibits the expression of BDNF by reducing the mRNA level of BDNF and its opposite function to BDNF [53]. BDNF-AS is downregulated in glioblastoma tissues and cells and interacts and stabilizes with the poly-adenosine-binding protein cytoplasm 1 (PABPC1) [54]. The overexpression of BDNF-AS inhibits the proliferation, migration, and invasion of glioblastoma cells and induces their apoptosis, while the effect of BDNF-AS knockout is opposite [55, 56].

As a research hotspot this year, lncRNA H19 has been proved to have a variety of biological functions, including regulating cell proliferation, differentiation, and metabolism [57]. It is also indirectly associated with the development of a number of other neurologic tumors, including medulloblastoma meningiomas and gliomas. In glioma cells, previous studies have demonstrated that lncRNA H19 is upregulated and promotes proliferation, migration, invasion, and angiogenesis through the miRNA-138/HIF-1 axis as ceRNA [58]. Meanwhile, both H19 and SOX4 are targets of miRNA-130a-3p. miRNA-130a is a carcinogenic miRNA that targets phosphatase and tensin homolog deleted on chromosome 10 (PTEN) to drive malignant cell survival and tumor growth [59]. Studies have demonstrated that SOX4 may lead to the occurrence of tumors and promote their epithelial-to-mesenchymal transition (EMT) [60]. There is a positive correlation between H19 and SOX4. In addition, SOX4 expression was significantly inhibited after H19 level was reduced [61]. The overexpression of H19 can significantly reduce the inhibitory effect of miRNA-130a-3P on SOX4.

The following table summarizes the CNS regulatory lncRNAs mentioned in this article (Table 1).

## 4. The Role of lncRNAs in Spinal Cord Nerve Injury Repair

Spinal cord injury (SCI) is a common traumatic disease, which often leads to permanent neurological defects. However, due to the limited understanding of the pathogenesis of SCI, there is still no effective treatment for this permanent neurological defect. In recent years, with the continuous recognition of lncRNAs by the scientific community, the regulatory role of lncRNAs in SCI has received more and more attention. Studies have demonstrated the role of lncRNAs in SCI pathogenesis, including neuronal loss of astrocyte proliferation and activation of microglia to activate inflammatory response [62].

Neuronal apoptosis is the main pathological feature of neuronal loss after spinal cord injury; neuronal autophagy can inhibit the apoptosis after spinal cord injury in rats to improve neuronal injury. Studies have demonstrated that TCTN2 overexpression in SCI rat models can reduce neuronal apoptosis by inducing autophagy, and it has been observed that TCTN2 negatively regulates miRNA-216b. Moreover, the miRNA-216b/Beclin-1 pathway can promote autophagy and inhibit cell apoptosis, thus alleviating spinal cord injury [63, 64]. In addition, lncRNA-Map 2k4 can also promote neuronal proliferation and inhibit neuronal apoptosis through the miRNA-199a/FGF1 pathway [65]. On the other hand, the study has demonstrated that inhibiting tumor regulatory factor lncRNA-IGF2AS can reverse the upward movement of IGF2 and increase the secretion of neurotrophic factors BDNF and NT3, promoting the growth of neurons [66].

Apoptosis of oligodendrocytes (OLs) and demyelination of surviving axons are significant components of SCI cascade secondary events, leading to nerve conduction failure [67]. Therefore, the promotion of remyelination is one of the significant factors to promote functional recovery after spinal cord injury. The overexpression of lncRNA OL1 promotes the differentiation of developing precocious oligocytes, while inactivation of lncRNA OL1 may lead to defects in the central nervous system myelin and remyelin after injury [68]. lncRNA OL1 promotes the maturation of oligos by binding to Suz12 as a complex [69].

Reactive astrocyte proliferation and glial hyperplasia are typical characteristics after spinal cord injury and can lead to the formation of glial scar, resulting in physical and biochemical impairments in plasticity and regeneration and ultimately inhibiting functional recovery [70]. However, reactive astrocytes are also beneficial factors for spinal cord injury, including endogenous neuroprotection and secretion of growth-promoting neurotrophic factor [71]. It was found that the downregulation of lncRNA SCIR1 may promote the proliferation and migration of astrocytes in vitro and may play an adverse role in the pathophysiology of SCI. In the model of acute contusion spinal cord injury, the downregulation of lncRNA SCIR1 was closely related to the decrease/increase of WNT3/BMP7 expression and the promotion of astrocyte proliferation and migration [72], suggesting that lncSCIR1 may be a beneficial factor for traumatic spinal cord injury [71]. Therefore, local overexpression of lncSCIR1 may help to neutralize the inhibitory environment around the lesion site and promote functional recovery. Another study showed that lncRNA Gm4419 can promote the apoptosis of trauma-induced astrocytes by upregulating the expression of inflammatory cytokine tumor necrosis factor-*α* (TNF-*α*), while the upregulation of TNF-*α* may be achieved by competitive binding of miRNA-466 [73].

Microglial inflammation is a significant biological process in response to injury, infection, and trauma suffered by cells or tissues. Activated microglia release a number of proinflammatory molecules such as interleukin-1 (IL-1B), TNF-reactive oxygen species, and nitric oxide [74]. After spinal cord injury, microglia cells experience significant changes in cell molecules and functions, and the activation of microglia cells is often used to represent neuronal inflammation in the secondary stage of spinal cord injury [62]. Studies have demonstrated that KLF4 is involved in the spinal cord injury process and can regulate the activation of microglia cells and subsequent neuroinflammation [75]. In SCI rat model, lncRNAs SNHG5 and KLF4 are highly expressed during SCI, which proves that KLF4 is the direct target of SNHG5 and is positively regulated by SNHG5. The study also proved that SNHG5 can enhance the activity of astrocytes and microglia cells and promote the process of spinal cord injury by upregulating KLF4 [76]. On the other hand, the overexpression of lncRNA TUSC7 has been demonstrated to inhibit microglial activation and inflammatory cytokine expression by regulating the expression of peroxisome proliferator-activated receptor (PPAR-) by miRNA-449a [77]. Meanwhile, as an epigenetic regulator of microglia cell polarization, lncRNA GAS5 can inhibit microglia cell M2 polarization [78]. Therefore, GAS5 is considered as a promising target for the treatment of demyelination diseases.

The release of a large number of inflammatory factors after spinal cord injury is also a common pathophysiological process of spinal cord injury [79]. It was found that the expression of TLR4 and leucine-rich repeats (TRIL) could be inhibited by downregulating lncRNA TUG1.TRIL is a receptor-assisted protein of TLR4 and plays a significant role in regulating the activity of TLR4 and its downstream inflammatory cytokine IL-1 [80]. Therefore, inhibition of lncRNAs TUG1 and further reduction of TLR4 expression reduce the release of inflammatory cytokines after spinal cord injury, especially after ischemia-reperfusion [81]. lncRNA LINC00341 is one of the most abundant lncRNAs in endothelial cells. Studies have demonstrated that lncRNA LINC00341 can inhibit the expression of vascular cell adhesion molecule 1 (VCAM1), inhibit mononuclear cell adhesion, and play an anti-inflammatory effect [82].

The following table summarizes the SCI regulatory lncRNAs mentioned in this article (Table 2).

## 5. The Role of lncRNAs in the Repair of Peripheral Nerve Injury

Peripheral nerve injury is a common neurological disease. The peripheral nervous system (PNS) differs from the central nervous system in that nerve regeneration is activated after peripheral neuron injury. Although the peripheral nervous system is capable of axonal regeneration, PNS often shows incomplete functional recovery after nerve injury. After peripheral nerve injury, a series of pathophysiological changes will occur at the site of injury, including the proliferation and migration of axial mutant Schwann cells (SCs) to form Büngner band, thus providing a suitable microenvironment and promoting axonal regeneration [83]. However, although peripheral neurons have the ability to regenerate, the molecular mechanisms of nerve regeneration in PNS have not been fully elucidated. So far, there is still no effective treatment for peripheral nerve injury. Therefore, it is significant to develop new and effective treatment methods to promote regeneration of peripheral nerve after injury. In recent years, many studies have found that ncRNAs, especially lncRNAs, are differentially expressed in peripheral nerve injury, which plays a significant regulatory role in nerve injury and regeneration.

Yu and Zhou indicated that in the rat model of sciatic nerve injury, according to the results of chip analysis and quantitative polymerase chain reaction verification, there were significant differences in the expression levels of various lncRNAs, among which Bc088327 indicated the highest upregulation. In addition, the knockout of lncRNA Bc088327 inhibits the SC vitality, inducing cell apoptosis and S-phase cell cycle arrest [84]. Therefore, lncRNA Bc088327 can be used as both a biomarker to detect the degree of nerve injury and a new therapeutic target to promote nerve repair.

Dorsal root ganglion (DRG) neurons, on the other hand, act as connections between peripheral tissue and the spinal cord. Transcriptional plasticity of DRG sensory neurons contributes to nerve repair after peripheral nerve injury, but also leads to maladaptive plasticity, including the development of neuropathic pain [85, 86]. Studies have demonstrated that lncRNA MRAK009713 is significantly upregulated in DRG induced by chronic sciatic nerve injury (CCI) in rats and participates in CCI-induced neuropathic pain by regulating the expression and function of DRG P2X3 receptor [86]. Meanwhile, lncRNA BC089918 inhibits the growth of DRG neuronal processes, and BC089918 can promote the growth of DRG neuronal processes in primary culture by siRNA knockout, while Fam57b Kcns1 and Cacng2 are potential targets of BC089918 [87].

The majority of peripheral nerve injury distal cells are Schwann cells whose dedifferentiation, proliferation, migration, and myelin removal are significantly associated with successful nerve regeneration. Blocking Schwann cell proliferation and migration may reduce axonal regeneration in transected nerves [88, 89].

The study has demonstrated that silencing lncRNA Tnax-PS1 can promote Schwann cell migration, and further study has demonstrated that TNXA-PS1 may reduce the inhibitory effect of dual-specificity phosphatase 1 (DUSP1) mediated by miRNA-24-3p/miRNA125-3P by sponging miRNA-24-3p/miRNA-125-3p [90]. After peripheral nerve injury, the expression of DUSP1 was decreased due to the downregulation of TNXA-PS1, which ultimately promoted the migration of Schwann cells. MEG3 is a tumor suppressor gene, which is downregulated in a variety of malignant tumors. Peripheral nerve injury is often accompanied by ischemia and inflammation, leading to the accumulation of reactive oxygen species (ROS) [91], leading to MEG3 upregulation [61]. Downregulation of lncRNA MEG3 promotes proliferation and migration of SCs through the PTEN/PI3K/AKT pathway, while silencing lncRNA MEG3 promotes the migration of SCs and the growth of axons and promotes nerve regeneration and functional recovery [92]. Studies have demonstrated that lncRNA BC088259 can interact with vimentin to regulate Schwann cell migration after compression injury of the sciatic nerve. Vimentin is a major intermediate filament protein involved in the developmental attachment and migration of cells. Silencing vimentin expression in Schwann cells significantly inhibited migration of Schwann cells [93]. In Pan and Shi's studies, the mRNA and protein levels of Ctrc1, Wnt5a, ROR2, Rac1, JNK, and ROCK were significantly upregulated, confirming that lncRNA NONMMUG014387 can promote proliferation of Schwann cells around the damaged site by targeting Ctrc1 and activating the Wnt/PCP pathway [94].

## 6. Regulatory Effect of lncRNAs on Neural-Like Differentiation of Stem Cells

Although more and more attention has been paid to the multidirectional differentiation of stem cell, the molecular regulation mechanism and influencing factors on the differentiation are still not clear. In recent years, with the continuous research and exploration of lncRNA, many lncRNAs have been found to play a significant regulatory role in the process of neural-like differentiation of stem cells.

Adipocyte differentiation-associated lncRNA (ADNCR) and TCF3 expression levels were decreased during the induction of differentiation of neural stem cells into nerve cells and astrocytes [95, 96]. However, the expression of miRNA-204-5p increased over time during the differentiation of neural stem cells into nerve cells and astrocytes [97]. As ceRNA of miRNA-204-5p, ADNCR overexpression inhibited the expression of NSC miRNA-204-5p and enhanced the expression of TCF3. Ectopic expression of ADNCR induces proliferation of NSCs and inhibits neuronal differentiation of NSCs, partly by regulating the expression of miRNA-204-5p/TCF3 [98].

**Figure 1:**
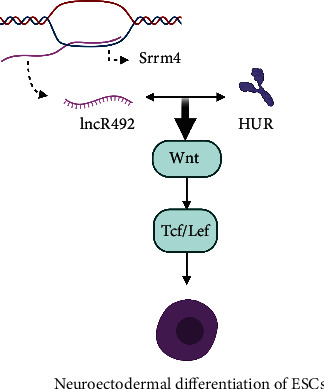
lncR492 interacts with HUR and participates in the maintenance of Wnt signaling, thus affecting the neuroectodermal differentiation of ESCs.

Winzi et al. demonstrated that lncRNA lncR492 can inhibit the differentiation of embryonic stem cells (ESC) into neurons [99]. lncR492 was located in the first intron of the protein-coding gene Srrm4, but the downregulation and overexpression of lncRNA did not affect the expression of Srrm4 [100]. Both lncR492 and HUR are involved in the maintenance of Wnt signaling, as the downregulation and overexpression of these two genes lead to the decrease and increase of Wnt signaling, respectively [99]. In vitro studies of ESC have demonstrated that members of the downstream effector Tcf/Lef protein family of Wnt signaling are significant in the differentiation process (Figure 1) [101, 102].

**Figure 2:**
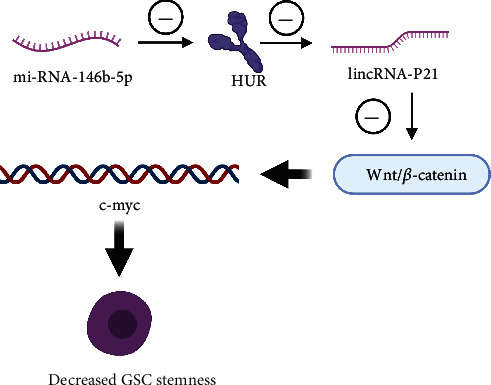
The downregulation of miRNA-146b-5p in GSCs leads to the upregulation of Hu antigen R (HUR) expression and then the downregulation of lincRNA-P21 expression and the upregulation of *β*-catenin expression, thus decreasing the stemness of GSCs.

Glioma stem cells (GSCs) have been demonstrated to be associated with glioma invasion, angiogenesis, immune evasion, and therapeutic resistance [103]. lincRNA-p21, a novel regulator of cell proliferation, apoptosis, and DNA damage, is downregulated in several types of tumors [104]. Studies have demonstrated that the downregulation of miRNA-146b-5p in GSCs leads to the upregulation of Hu antigen R (HUR) expression and then the downregulation of lincRNA-P21 expression and the upregulation of *β*-catenin expression. miRNA-146b-5p inhibit the proliferation of gliomas and thus promote the apoptosis of gliomas [105]. Overexpression of miRNA-146b-5p also increases the apoptosis and radiosensitivity of GSCs, reduces the expression of cell viability, neurogenesis, and stem cell markers, and induces the differentiation of GSCs [106]. In addition, downregulation of lincRNA-P21 or the overexpression of HUR and *β*-catenin saves the phenotypic changes induced by the overexpression of miRNA-146b-5p (Figure 2) [105]. These results suggest that targeting the miRNA-146b-5p/HUR/lincRNA-p21/-catenin signaling pathway may be a valuable treatment strategy for glioma.

The following table summarizes the PNS and stem cell regulatory lncRNAs mentioned in this article (Table 3).

## 7. Concluding Remarks

This paper mainly describes the regulatory role of lncRNAs in various nervous systems and in neural differentiation of stem cells in various physiological or pathological processes. lncRNA is significant and cannot be ignored in molecular mechanisms of growth, development, and injury repair. In the process of writing this article, we also found that, at present, most research senior lncRNA focus on growth and development of the central nervous system, brain, neurodegenerative diseases, and spinal cord injury, although there are relatively few studies of the peripheral nervous system injury and differentiation of neural stem cells, so in the future study, lncRNA system around nerve injury, rehabilitation, and neural stem cells differentiating the regulating mechanism in the process will be the research foreground and research value. In addition, most of the existing studies have not described in detail the regulatory mechanism of lncRNA, so it is also a significant research direction to explore the specific mechanism of lncRNA in regulating the activity of the nervous system.

In general, more and more studies have revealed the key role and related mechanism of lncRNAs in the development, growth, and regeneration of nervous systems. With more studies and discoveries, lncRNAs are likely to become a significant therapeutic target for the treatment of various neurological diseases in the future.

## Figures and Tables

**Table 1:** lncRNAs which regulate CNS mentioned in this article.

lncRNA	Effection	Mechanism
Pnky [26]	Regulates the neurogenesis of embryonic and postnatal brain neural stem cells	Pnky interacts with PTBP1 and regulates the neurogenesis of embryonic and postnatal brain neural stem cells.
Evf2 [28]	Participates in the differentiation of GABA cells	Evf2 recruits DLX and MECP2 transcription factors and controls the expression of D1x5, D1x6, and GAD67.
Six30S [34]	Regulates early eye formation and postnatal retinal cells	Six30S can directly combine known Six3 transcription-assisted regulators and histone-modifying enzymes and act as an RNA-based transcription scaffold.
Paupar [35, 37]	The generation of retinal neurons and the control of the differentiation of a variety of late neuron cell types	By forming ribonucleoprotein complexes containing KAP1 and PAX6 transcription factors.
NRON [40]	Involved in the development of certain disorders of DS	DSCR1 and DYRK1A expression was upregulated and NFATc activity was decreased.
BACE1 [42]	Participates in the occurrence of AD	The mechanism is unclear.
DGCRS [44]	Increases the risk of SCZ through its regulatory effect on coexpressed SCZ-related genes	The mechanism is unclear.
HOTAIR [45, 46]	Participates in the autophagy of dopaminergic neurons in the dense region of the substantia nigra	HOTAIR can bind to miRNA-221-3P and improve the expression of NPTX2, thus reducing cell activity and enhancing the autophagy ability of dopaminergic neurons.
n333955, n332943, n335470, ENST00000384390, and n341115 [47, 48]	The downregulation is significant in TBI	The mechanism is unclear.
TCONS 00018733-XLOC 008489, 0TTHUMT00000076953, NR029967, ENST00000433249, and n381234 [47, 48]	The upregulation was significant in TBI	The mechanism is unclear.
BDNF-AS [54]	Inhibits the proliferation, migration, and invasion of glioblastoma cells	Reduces mRNA level of BDNF and inhibits the expression of BDNF
H19 [59]	Drives the survival of malignant cells, the growth of tumor, and the transformation of dermal stroma	miRNA-130a-3p was upregulated. PTEN was targeted, and the inhibitory effect of miRNA-130a-3P on SOX4 was weakened.

**Table 2:** lncRNAs which regulate SCI mentioned in this article.

lncRNA	Effection	Mechanism
TCTN2 [63, 64]	Induction of autophagy reduces neuronal apoptosis	Negative regulation of miR-216b, promoted autophagy and inhibited apoptosis
Map2k4 [65]	Promotes neuronal proliferation and inhibits neuronal apoptosis	The miR-199a/FGF1 pathway promoted neuronal proliferation and inhibited neuronal apoptosis.
IGF2AS [66]	Promotes neuronal growth	Upregulate IGF2 and increase the secretion of neurotrophic factors BDNF and NT3
Inc0L1 [68]	Promotes the differentiation of developing precocious oligodendrocytes	The mechanism is unclear.
SCIR1 [72]	Participates in the proliferation and migration of astrocytes in vitro	The downregulation of IncSCIR1 decreased/increased WNT3/BMP7 expression and promoted the proliferation and migration of astrocytes.
Gm4419 [73]	Participates in the apoptosis of astrocytes	Upregulation of inflammatory cytokines tumor necrosis factor-*α* (TNF-*α*) by competitive binding of miR-466
SNHG5 [76]	Regulates microglia cell activation and subsequent neuroinflammation	By upregulating KLF4, the activity of astrocytes and microglia cells was increased and the progression of spinal cord injury was promoted.
TUSC7 [77]	Inhibition of microglia activation and inflammatory cytokines expression	The expression of peroxisome proliferator-activated receptor *γ*(PPAR-*γ*) was regulated by miR-449a
GAS5 [78]	Inhibits M2 polarization of microglia cells	The mechanism is unclear.
TUG1 [80]	Involved the release of inflammatory cytokines after spinal cord injury, especially after ischemia-reperfusion	Inhibits lncRNA TUG1 and decreases TLR4 expression and its downstream inflammatory cytokine IL-1
LINC00341 [82]	Anti-inflammatory effects	Inhibits vascular cell adhesion molecule 1 (VCAM1) expression

**Table 3:** lncRNAs which regulate PNS and stem cells mentioned in this article.

lncRNA	Effection	Mechanism
BC088327 [84]	As a biomarker to detect the degree of nerve damage	Inhibits SC cell vitality, induces apoptosis and S-phase cell cycle arrest
MRAK009713 [86]	Participates in ccl-induced neuropathic pain	Regulating the expression and function of DRG P2X3 receptor participates in ccl-induced neuropathic pain
BC089918 [87]	Inhibits the growth of DRG neuronal processes	The mechanism is unclear.
TNAX-PS1 [90]	Regulates Schwann cell migration	Sponging miR-24-3p/miR-125-3p attenuated the inhibition of bispecific phosphatase 1 (DUSP1) mediated by miR-24-3p/miR-125-3p
MEG3 [92]	Promotes SC proliferation and migration	Silencing lncRNA MEG3, promotes SC migration and axonal growth through the PTEN/P13K/AKT pathway
BC088259 [93]	Regulates migration of Schwann cells	Interacts with vimentin
N0NMMUG0148387 [94]	Promotes the proliferation of Schwann cells around the damaged site	Targeted regulation of Ctrc1 and activation of the Wnt/PCP pathway
ADNCR [98]	Participates in the differentiation of NSCs into neurons	Overexpression of ANDCR inhibited the expression of NSC miR-204-5p and enhanced the expression of TCF3.
lncR492 [99]	Inhibits the differentiation of embryonic stem cells (ESC) into neurons	The expression level of lncR492 regulates the Wnt signaling pathway and members of its downstream effector Tcf/Lef protein family.
lincRNA-p21 [105]	Participates in the proliferation and apoptosis of glioma	Involved in the regulation of the miR-146b-5p/HUR/lincRNA-P21/-catenin signaling pathway

## Data Availability

The reference data supporting this systematic review are from previously reported studies and datasets, which have been cited. The processed data are available at PubMed.
